# Printing, Debinding and Sintering of 15-5PH Stainless Steel Components by Fused Deposition Modeling Additive Manufacturing

**DOI:** 10.3390/ma16196372

**Published:** 2023-09-23

**Authors:** Gaoyuan Chang, Xiaoxun Zhang, Fang Ma, Cheng Zhang, Luyang Xu

**Affiliations:** 1 School of Materials Science and Engineering, Shanghai University of Engineering Science, Shanghai 201620, China; 2 School of Mechanical and Automotive Engineering, Shanghai University of Engineering Science, Shanghai 201620, China

**Keywords:** metal fused deposition modeling, additive manufacturing, 15-5PH stainless steel, debinding, sintering

## Abstract

Metal FDM technology overcomes the problems of high cost, high energy consumption and high material requirements of traditional metal additive manufacturing by combining FDM and powder metallurgy and realizes the low-cost manufacturing of complex metal parts. In this work, 15-5PH stainless steel granules with a powder content of 90% and suitable for metal FDM were developed. The flowability and formability of the feedstock were investigated and the parts were printed. A two-step (solvent and thermal) debinding process is used to remove the binder from the green part. After being kept at 75 °C in cyclohexane for 24 h, the solvent debinding rate reached 98.7%. Following thermal debinding, the material’s weight decreased by slightly more than 10%. Sintering was conducted at 1300 °C, 1375 °C and 1390 °C in a hydrogen atmosphere. The results show that the shrinkage of the sintered components in the X-Y-Z direction remains quite consistent, with values ranging from 13.26% to 19.58% between 1300 °C and 1390 °C. After sintering at 1390 °C, the material exhibited a relative density of 95.83%, a hardness of 101.63 HRBW and a remarkable tensile strength of 770 MPa. This work realizes the production of metal parts using 15-5PH granules’ extrusion additive manufacturing, providing a method for the low-cost preparation of metal parts. And it provides a useful reference for the debinding and sintering process settings of metal FDM. In addition, it also enriches the selection range of materials for metal FDM.

## 1. Introduction

Additive manufacturing (AM) stands as a groundbreaking technology that utilizes computer-aided design models to construct products by adding material layer by layer. Unlike traditional manufacturing methods, AM allows for the creation of products with diverse materials, complex shapes, structures and functions, without the need for subsequent post-processing such as cutting and machining [1]. The common materials used in AM include metals, ceramics and plastics, and the choice of AM method varies depending on the specific material being used [2]. At present, predominant metal AM techniques encompass selective laser melting (SLM) [3,4,5], selective laser sintering (SLS) [6,7], electron beam melting (EBM) [8,9], direct laser metal sintering (DLMF) [10], binder injection (BJ) [11], wire arc additive manufacturing (WAAM) [12] and laser bed powder fusion (LBPF) [13]. Most of these techniques use lasers to subject the material to a process of high temperature and rapid cooling. However, the high temperature gradients and rapid cooling under these processes cause material anisotropy and generate high residual stresses that affect the mechanical properties of the material [14,15,16,17]. In addition, it is too expensive to purchase this equipment, with high maintenance and repair costs, which require a great quantity of upfront investment. For example, SLM and EBM printers require laser or electron beams and inert or vacuum chambers, and WAAM technology preparation requires high-precision equipment, which is complex and expensive. Therefore, developing a low-cost metal AM method has great practical significance. Fused deposition molding (FDM) emerges as a promising low-cost AM technology that operates at room temperature. The material at the nozzle is heated to the melting temperature (typically between 200 °C and 300 °C), without complex energy sources. This makes FDM equipment much cheaper than equipment such as SLM. The process involves heating and melting a hot molten material, which is then extruded through a printing nozzle. The nozzle moves along a specific trajectory under computer control, depositing the material in a semi-fluid state onto a printing platform. The material solidifies and forms the final solid product by stacking layers. Another technique, known as metal injection molding (MIM), involves injecting a mixture of metal powder and polymer into a molding chamber to achieve the desired shape [18,19]. The polymer is then removed through a debinding process, followed by sintering the metal powder below its melting point to obtain densely consolidated metal parts. By incorporating the pellets and post-processing techniques used in MIM into FDM, it is possible to combine the advantages of both technologies. This allows for the production of complex structural parts using FDM, a low-cost technology, while achieving dense metal parts through the debinding and sintering processes. In this process, the printed part is often referred to as the “green part”, the solvent debinded part is called the “brown part”, and the sintered part is called the “FDMS part”. The feedstock used in metal FDM technology is a mixture of metal powder and a specified proportion of polymer material, usually in the form of 1.75 mm diameter wire or approximately 3 mm diameter pellets. The fluidity of the material is provided by the polymer in the feedstock, which only needs to be heated at the nozzle for smooth extrusion. Metal powders and polymers are readily available, and the pellet feedstock for this process can be prepared at a cost of as little as 20 USD/kg. The printing, debinding and sintering equipment used in the metal FDM is cheaper and less expensive to maintain than those requiring electronics and lasers. These features of metal FDM significantly reduce the cost of producing metal parts by AM, making it suitable for large-scale use in laboratories and small businesses.

Many scholars have shown great interest in metal FDM in recent years. Yvonne Thompson et al. [20] prepared printable FFF filaments from grafted polyolefin, thermoplastic elastomer and 55 vol.% 316L powder and optimized printing parameters, particularly studying the debinding and sintering process, resulting in 316L specimens with a shrinkage of about 20% and a relative density of more than 95%. M. Sadaf [21] developed filaments containing 65 vol.% of 316L steel powder using a one-component binder, which avoided the solvent debinding process. This was followed by sintering the green parts at 1380 °C under hydrogen to obtain metal specimens with a tensile strength of 520 MPa and a hardness of 285 HV. Liu Bin et al. [22] prepared 316L/POM filaments and used catalytic debinding to remove the binder, studied the microstructural characteristics of the filaments, green parts and sintered parts and tested the relative density, tensile properties, hardness and shrinkage of the sintered parts. Gurminder Singh et al. [23] investigated commercial MIM Cu raw materials using screw-type printers. They delved into the impact of layer thickness, nozzle temperature, extrusion multiplicity and printing speed on the density and surface roughness of green parts. Utilizing a central composite design approach, they assessed these factors and employed micro-tomography to examine sample porosity under various process parameters. The optimised green parts were also sintered to obtain high-density sintered copper parts with a low surface roughness. A high metal powder content results in a low shrinkage of the sintered parts. The researchers therefore expect to be able to increase the metal powder content as much as possible while maintaining normal printing conditions. Materials with a relatively high metal powder content have a poor flowability, and preparing filaments that can pass through the printer rollers without breaking is a difficult task. Furthermore, the preparation of filaments is already a lengthy process compared to granules, prompting researchers to explore screw printers as an alternative solution. Similarly, Gurminder Singh et al. [24] used the MIM17-4PH feedstock for printing under optimum parameters to investigate the density of the sintered parts at different temperatures. Qualitative and quantitative analysis of the pores in the green and sintered parts using micro-tomography confirmed that the optimized printing parameters were beneficial for the final microstructure. In addition, several researchers have also investigated the FDM process of other metallic or ceramic materials; for example, 316L [25,26,27], 17-4PH [28,29], copper [30,31], hardmetal [32], Ti6Al4V [33,34], tungsten heavy alloy [35], H13 [36], Al [37], 1.2083 steel [38], zirconia [39,40], etc. However, no studies on the FDM of 15-5PH stainless steel (15-5PH SS) materials were found in the available literature.

The 15-5PH stainless steel, derived from 17-4PH steel through a reduction in Cr content and increase in Ni content, represents a martensitic precipitation-hardening stainless steel renowned for its exceptional mechanical properties and corrosion resistance [41]. Therefore, it finds widespread use as an engineering material in aerospace, medical, chemical and other fields [42,43,44]. At the same time, 3D printing is gradually maturing, and the application scenarios are constantly expanding. In this work, we developed a 15-5PH granular feedstock with a metal powder content of up to 90 wt.% and successfully constructed parts using a screw-type printer and produced metal parts after debinding and sintering. Firstly, this research delved into the impact of flowability and the printing process parameters of 15-5PH granular material on the forming state of the parts. Furthermore, we explored both solvent and thermal debinding processes. Subsequently, this work investigated the influence of sintering temperature on the densities, shrinkage, mechanical properties and micromorphology of the sintered parts. Aiming at the current problems of metal AM with expensive equipment, complicated operation, and mainly oriented to high-precision enterprises, this study aims to explore a low-cost metal additive manufacturing technology that is simple to operate and easy to popularize. This work not only achieves low-cost metal AM but also expands the range of materials used for metal FDM.

## 2. Experimental Details

**Figure 1:**
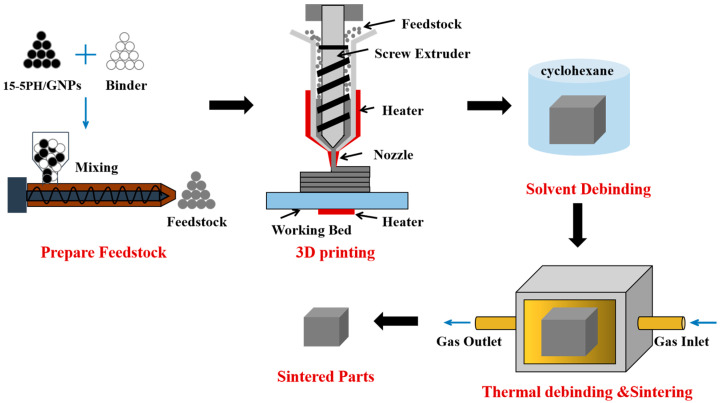
Process flow diagram of 15-5PH stainless steel by metal FDM.

The process flow diagram of this work is depicted in Figure 1. Detailed elaboration of the specific steps will be provided in the subsequent sections.

### 2.1. Materials and Preparation of Feedstock

**Figure 2:**
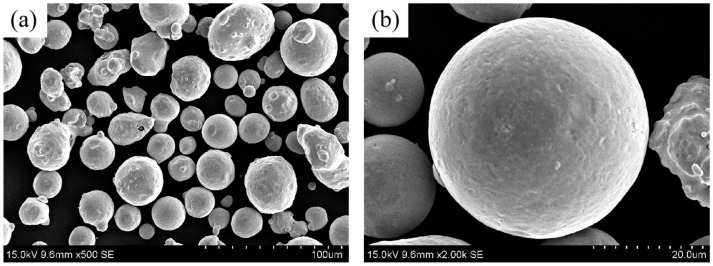
SEM image of 15-5PH stainless steel powder: (**a**) with magnifying powder of 500×, (**b**) with magnifying powder of 2000×.

The metal powder employed in this investigation is a gas-atomized 15-5PH SS powder with a sphericity of 96%, supplied by PMG 3D Technologies (Shanghai) Co., Ltd. (Shanghai, China). The powder particle size is within the range of 15–45 μm. All illustration of the powder’s morphology is presented in Figure 2. The chemical composition of 15-5PH SS is provided by the distributor as shown in Table 1. Thermoplastic elastomer (TPE) was selected as the soluble binder and maleic anhydride grafted polypropylene (MAH-g-PP) as the insoluble backbone. Both binders were purchased from Dongguan Qihong Plastic Co., Ltd. (Dongguan, China). The MAH-g-PP was produced by introducing a strong polar maleic anhydride side branch into the main chain of the non-polar molecules of polypropylene, which can enhance the compatibility of polar and non-polar materials and facilitate the dispersion of metal powders. None of the above raw materials were further treated before the experiment. The proportions of metal powders and binder compositions used in this work are given in Table 2.

The 15-5PH granules that can be used for 3D printing were prepared as follows: 15-5PH stainless steel powder and the binder were put into a high-speed mixer for mixing according to the ratio in the Table 2. The mixed material was fed by the conical twin-screw extruder for compound extrusion. The twin-screw extruder was maintained at temperatures of 190 °C, 195 °C, 195 °C, and 200 °C in each respective zone, while the extrusion speed was set as 25 rpm. The extruded material was subsequently granulated using a granulator. To ensure the even distribution of the metal powder in the binder, the granules were again extruded in the extruder. The reextruded particles are placed in a vacuum drying oven for 3 h and stored in a vacuum environment for later printing.

### 2.2. 3D Printing

**Figure 3:**
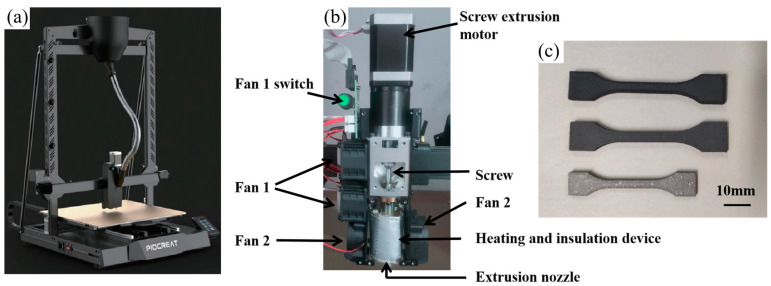
(**a**) G5Pro printer, (**b**) the structure of G5Pro printer and (**c**) the parts after being printed (up), debinded (middle) and sintered (down).

The part was initially designed in Solidworks (version number: 2021) software and then converted to the STL format. Subsequently, the model underwent slicing using Simplify3D software (version number: 4.0.1) and was imported into a G5 Pro printer from Shenzhen Piocreat 3D Technology Co., Ltd. (Shenzhen, China). The printer is a screw-type printer, which can print granular materials directly, avoiding the process of filament preparation. Its morphology and structure are shown in Figure 3a,b. The printer transports particles through a screw movement to a heating unit, where the material is melted and extruded through nozzles to be deposited on the platform. The printer platform is a metal substrate with a good thermal conductivity that heats up quickly. The machine has a print size of 500 × 500 × 500 mm and a print speed range of 0–100 mm/s. The slice thickness is 0.1–1 mm and the positioning accuracy of the X-Y axis is ±0.1 mm, allowing the production of complex geometries. In this study, dog bone tensile specimens (length 66 mm, thickness 3 mm, maximum width 12 mm, minimum width 4 mm) and small blocks (15 mm × 15 mm × 3 mm) were printed. Figure 3c presents the morphology of the printed, solvent debinded and sintered part. To match the material’s flow characteristics, a nozzle diameter of 0.8 mm was selected. The printing parameters were set as follows: fill rate 100%, flow rate multiplier 180%, layer thickness 0.2 mm, printing speed 40 mm/s, nozzle temperature 285 °C, platform temperature 90 °C and fill angle ±45°. The nozzle fan was switched off during the printing process.

### 2.3. Debinding and Sintering

The debinding process consists of solvent debinding and thermal debinding. Solvent debinding removes the soluble binder TPE. The parts were immersed in cyclohexane at 25 °C, 45 °C, 65 °C and 75 °C for 24 h while being palced in a constant temperature magnetic stirrer. The parts were removed every two hours, then dried at 80 °C for 3 h within a vacuum oven before weighed. The rate of binder removal during solvent debinding was evaluated by calculating the ratio of binder loss after solvent debinding to the total binder. The ratio of weight loss in each step to the total weight was used to evaluate the binder removal rate throughout the debinding process.

**Figure 4:**
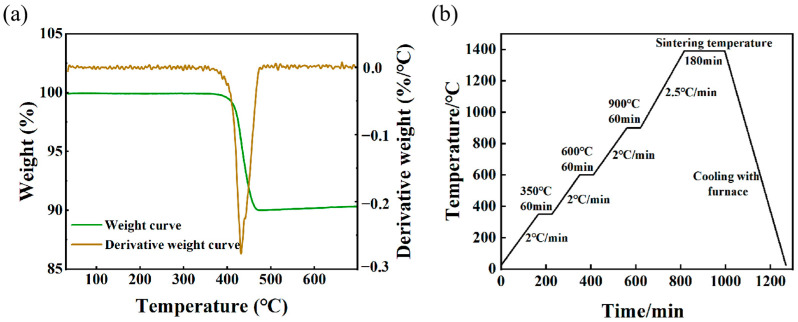
(**a**) TGA of 15-5PH granular feedstock; (**b**) thermal debinding and sintering curves.

Thermal debinding and sintering were conducted in a tube furnace (Shanghai Shiheng Instrument Equipment Co., Ltd. (Shanghai, China)), where the samples were heated to 1300 °C, 1375 °C and 1390 °C, respectively, and held for 3 h. Hydrogen gas was introduced at a flow rate of 250 mL/min. Thermal debinding removes the backbone binder from the sample and ends the debinding process. To establish the thermal debinding curve, we performed thermogravimetric analysis (TGA) on the granules. The TGA results, depicted in Figure 4a, were obtained in a nitrogen atmosphere with a heating rate of 10 °C/min, ranging from 30 °C to 700 °C. The results showed that the material started to decompose at 365 °C and finished at 471 °C. The green parts are heated to 600 °C and held at 350 °C and 600 °C to ensure complete binder removal during the thermal debinding. The heating rates applied for both thermal debinding and sintering were 2.0 °C/min and 2.5 °C/min, respectively. After insulation at the sintering temperature, the samples were cooled within the furnace. The corresponding thermal debinding and sintering curves are shown in Figure 4b.

### 2.4. Characterization and Test

In this study, an S-3400N scanning electron microscope (SEM) was used to observe the morphology of the granules, green parts, brown parts and sintered parts. The flowability of 15-5PH granules was tested at 265 °C, 275 °C, 285 °C and 295 °C using a capillary rheometer with a load of 21.6 kg. For the determination of sintered part density, we utilized Archimedes’ principle, repeating each measurement three times to ensure experimental precision. The sintered parts were corroded with aqua regia to observe the grain morphology and porosity. To calculate the shrinkage of the parts, the dimensions of the parts before and after sintering were measured with vernier calipers. Furthermore, the mechanical properties were evaluated through hardness and tensile tests on the sintered components, aiming to investigate the influence of sintering temperature. Sintered parts were subjected to hardness assessment using a Rockwell hardness tester, with ten measurements taken per sample, and the average value determined as the final hardness value. Tensile tests were conducted at room temperature, employing an AG-25TA tensile tester operating at a speed of 1 mm/min. Three tensile specimens were prepared under each sintering temperature to reduce the experimental error, and the results were averaged.

## 3. Results and Discussion

### 3.1. Feedstock Characterization and 3D Printing

**Figure 5:**
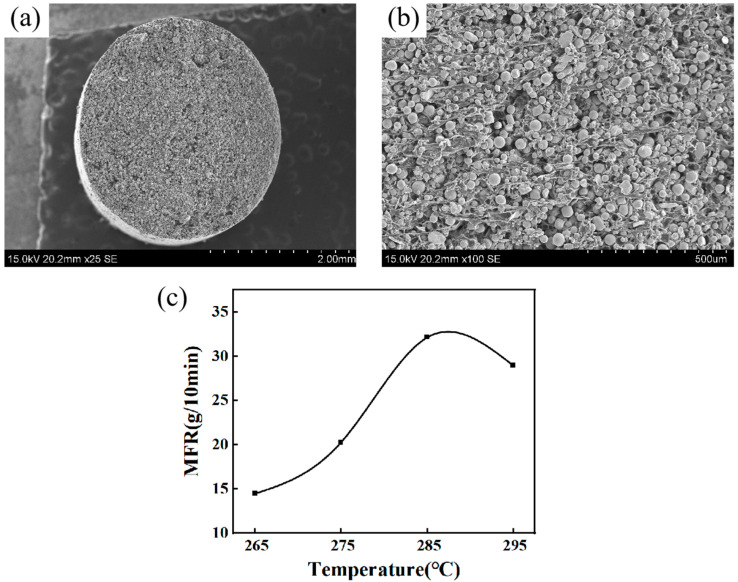
SEM image of 15-5PH granular feedstock: (**a**) 25×, (**b**) 100×; (**c**) melt mass flow rate of 15-5PH granular feedstock.

The microscopic morphology of the 15-5PH granular feedstock is depicted in Figure 5. Figure 5a,b illustrate that the metal powders are uniformly distributed between the binders without agglomeration. The homogeneous mixing of the metal powder and binder can prevent visible defects in the sintered parts and avoid warping and cracking [45]. The printing quality is closely related to the flowability of the feedstock [20,23]. Melt mass flow rate (MFR) measurements were performed on the granules to determine the nozzle temperature, and the results are presented in Figure 5c. The result indicates that the MFR of the feedstock exhibits sensitivity to temperature variations. As the temperature rises, the MFR tends to increase and then decrease, reaching the optimum value at 285 °C. Practice has also shown that the material extrusion process is extremely smooth when the nozzle temperature is 285 °C.

The 0.6 mm diameter nozzle often clogged and could not print normally. After changing the nozzle diameter to 0.8 mm, the printer extruded smoothly. The results of matching the flowability of the feedstock to the printer showed that when the extrusion multiplier was 100%, the material was under-extruded, resulting in parts that could not be moulded. Adjusting the extrusion multiplier to 180% resulted in a good part-molding quality. It was found that high-surface-quality parts could be obtained by switching off the fan at the nozzle during the printing process.

**Figure 6:**
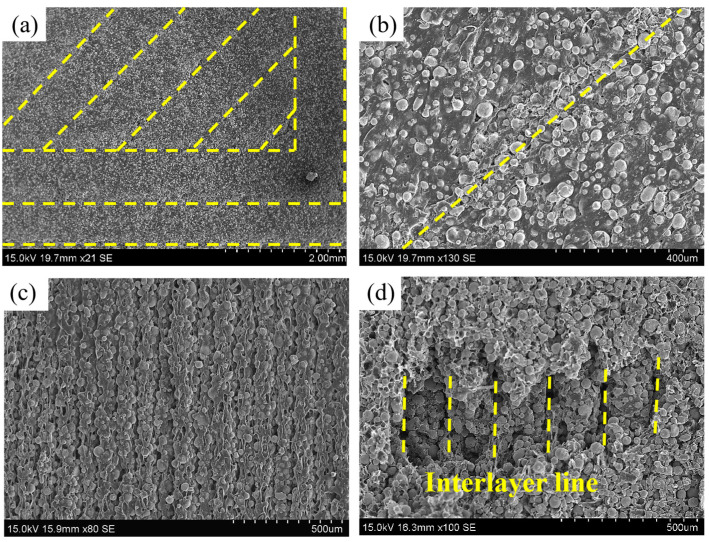
(**a**,**b**) Microscopic morphology of the green part, (**c**,**d**) side surface of the green part with magnifications of 80× and 100×.

Figure 6 presents the microscopic topography of the green part. Figure 6a,b show that the metal powder is still uniformly distributed in the binder after printing with the screw-type printer. Figure 6c,d show the layer thickness of the green part and the interlayer line during interlayer bonding, which is caused by the characteristics of FDM. The formation of interlayer lines introduces pores into the parts, which can impact the densities and mechanical properties of the sintered parts. The interlayer bonding effect can be modified by adjusting the printing parameters, thereby changing the mechanical properties of the parts [23,29,46].

### 3.2. Solvent and Thermal Debinding

**Figure 7:**
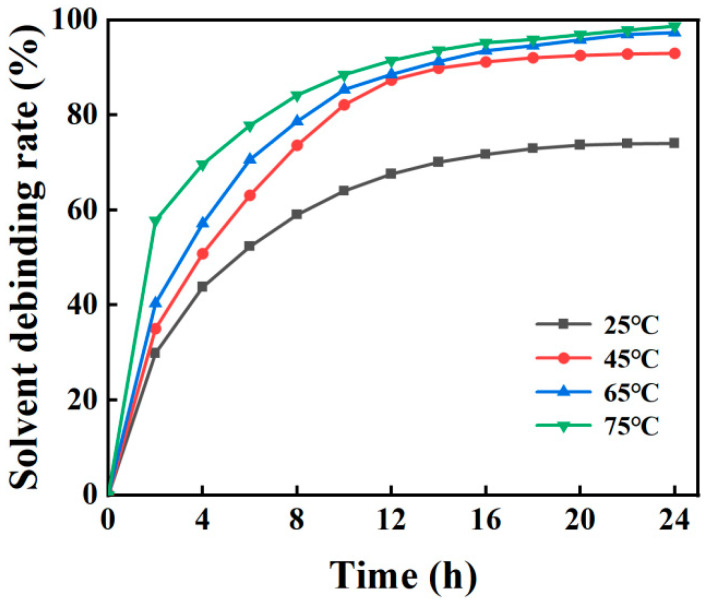
Solvent debinding rate for the green part at different temperatures and times.

**Figure 8:**
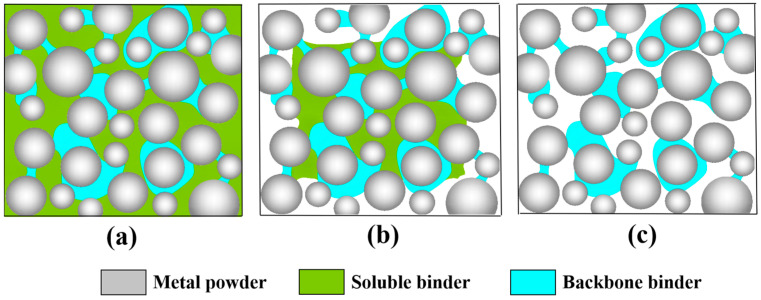
Schematic diagram of solvent debinding process. (**a**) the initial stage of solvent debinding, (**b**) the middle stage of solvent debinding; (**c**) the final stage of solvent debinding.

**Figure 9:**
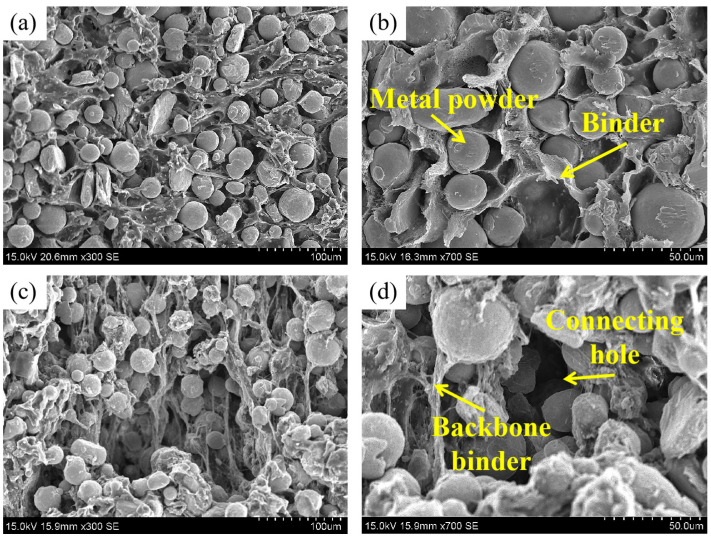
Microstructure of green part: (**a**,**b**) before solvent debinding, (**c**,**d**) after solvent debinding.

TPE is soluble in cyclohexane, and solvent debinding is used to remove soluble binders from the print [26]. The green parts were immersed in cyclohexane at different temperatures and kept for different times to investigate the effect of the debinding temperature and time on the solvent debinding rate, and the results are shown in Figure 7. The debinding rate increment of the parts decreased continuously over time at four temperatures. On the contrary, the debinding rate increased continuously over time, and the change in debinding rate was not significant after 14 h. The solvent debinding process mainly involves dissolution and diffusion, and the concentration of the solution and the distance of the solution diffusion path will affect the debinding rate increment. Figure 8 is a schematic diagram of the solvent debinding process. At the initial stage of solvent debinding (Figure 8a), the solution is in contact with the surface of the parts, the diffusion path is the shortest, and the solution concentration is the highest. As shown in Figure 7, the debinding rate increases from 0 to 57.83% in 2 h at a temperature of 75 °C. In the middle stage of solvent debinding, both the solution concentration and the diffusion path increase with time, as shown in Figure 8b, leading to a reduction in the debinding rate increment, although the debinding rate continues to rise. By the final stage of solvent debinding, the soluble binder is almost completely removed. Only PP remains in the printed parts, maintaining their shape, as shown in Figure 8c. The debinding rate increases with the increasing temperature for the same time, because the dissolution and diffusion velocity increase with increasing temperature. In addition, the solubility of cyclohexane increases at higher temperatures, which can dissolve more soluble components. The samples after solvent debinding (Figure 9) showed that pores had formed inside the parts and connecting holes had formed in some areas. These pores will provide channels for gas to escape during the thermal debinding process, preventing the bulging and cracking of the part [20].

**Figure 10:**
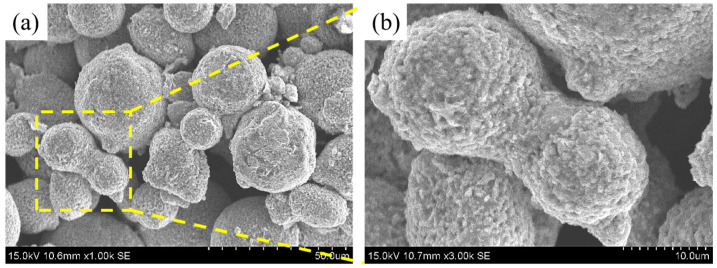
SEM morphology of the parts after thermal debinding and pre-sintering. (**a**) the sintering neck between the metal powder, (**b**) left image magnified 3000×.

The strength of the samples after thermal debinding proved to be insufficient for testing purposes. Therefore, the brown part was heated to 900 °C for pre-sintering to investigate the thermal debinding process. Figure 10 presents the morphology of the part after this process. After thermal debinding and pre-sintering, the binder has completely disappeared from the brown part and a sintering neck is formed between most of the metal particles. Table 3 provides the results of the total weight loss observed in the green parts after debinding. The binder mass in the granules is 10% of the total mass of the feedstock. The soluble components account for 7% and the backbone components account for 3% of the binder. Table 3 reveals that solvent debinding successfully removed the majority of the soluble binder from the green parts. Furthermore, after thermal debinding, both the backbone and any residual soluble binder were entirely eliminated. After debinding, the metal particles on the surface of the sample were lost during transport, resulting in a total weight loss of just over 10% of the feedstock.

### 3.3. Sintered Parts: Shrinkage, Relative Density, Microstructure and Mechanical Properties

**Figure 11:**
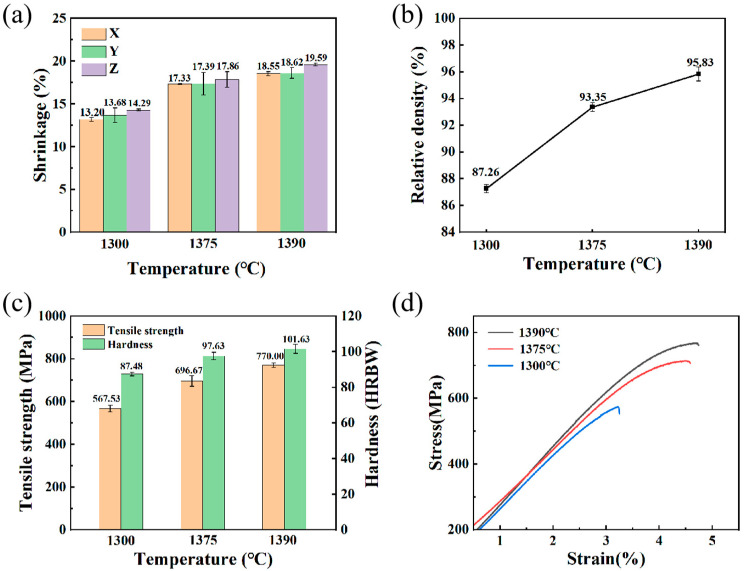
(**a**) Effect of sintering temperature on dimensional shrinkage, (**b**) effect of sintering temperature on relative density, (**c**) effect of sintering temperature on tensile strength and hardness, (**d**) stress–strain curve of the sintered parts.

The brown part undergoes high-temperature sintering to yield a densely consolidated metal component. Notably, brown parts sintered at different temperatures exhibit an excellent appearance, devoid of defects such as warping, collapse, bulging and cracking. Figure 11a illustrates the shrinkage observed in brown parts after sintering at 1300 °C, 1375 °C and 1390 °C for 3 h. The shrinkage of the sintered parts in the X-Y-Z direction remains relatively consistent at the same sintering temperature. The model can be scaled up accordingly during slicing to compensate for the dimensional shrinkage that occurs after sintering. As the temperature increases, the atomic migration velocity and the growth velocity of sintering necks increase, and the grain boundary growth velocity across the pores increases. Consequently, the internal pore volume within the part decreases compared to low-temperature sintering, ultimately resulting in an increase in dimensional shrinkage for the sintered part as the temperature rises.

The relationship between relative density and sintering temperature is illustrated in Figure 11b. As the sintering temperature rises, there is a corresponding increase in the relative density of the sintered part, mirroring the trend observed in dimensional shrinkage with temperature. The relative density of the sintered part reached 95.83% at 1390 °C, a figure comparable to that of sintered parts produced via MIM [47].

**Figure 12:**
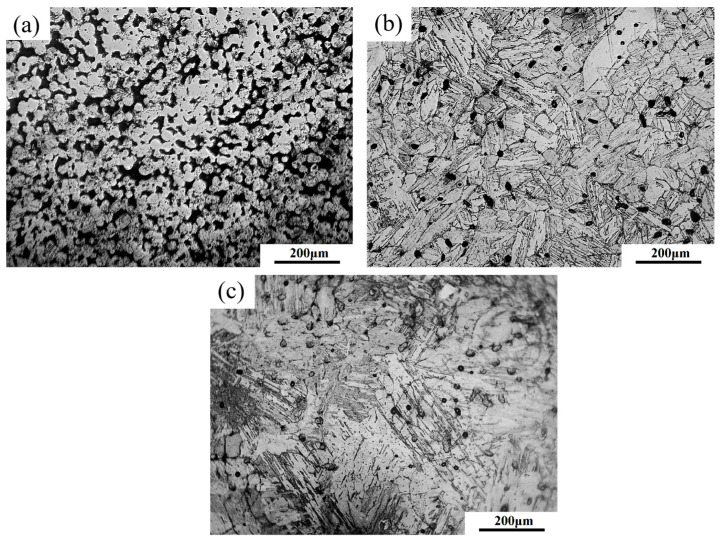
Microstructure of the parts sintered at (**a**) 1300 °C, (**b**) 1375 °C, (**c**) 1390 °C.

Figure 12 provides the microstructure of sintered parts at various temperature levels. When sintered at 1300 °C (Figure 12a), the number of pores in sintered parts was high and the size of the pores was large. Although the sintering neck between the powders grew with increasing temperatures between 900 °C and 1300 °C, the density was still low. There was no obvious grain morphology inside the parts after aqua regia corrosion. Following sintering at 1375 °C, there was a marked reduction in the number of pores within the samples, and the remaining pores tended to be mostly spherical and smaller in size. It can be seen that the structure is martensite after corrosion. With a further increase in temperature from 1375 °C to 1390 °C, both the number and size of pores continued to decrease. However, Figure 12c shows a increase in grain size.

The trend of the effect of sintering temperature on hardness and tensile strength is shown in Figure 11c. Both hardness and tensile strength increase with increasing sintering temperature, which is consistent with the trend of density with sintering temperature. Rockwell hardness is measured by pressing an indenter into the surface of an object with a certain force and determining the hardness from the depth of the indentation residue. The part sintered at 1300 °C contained a 12.74% porosity, which was easier for the indenter to press into during the hardness test, leaving deeper craters in the samples, resulting in a relatively low hardness of 87.48 ± 1.03 HRBW. After sintering at 1390 °C, the parts had a porosity of 4.17% and a high density. The dense structure impedes the indenter during the testing process, resulting in a shallower indentation depth remaining on the surface and a corresponding increase in hardness.

**Figure 13:**
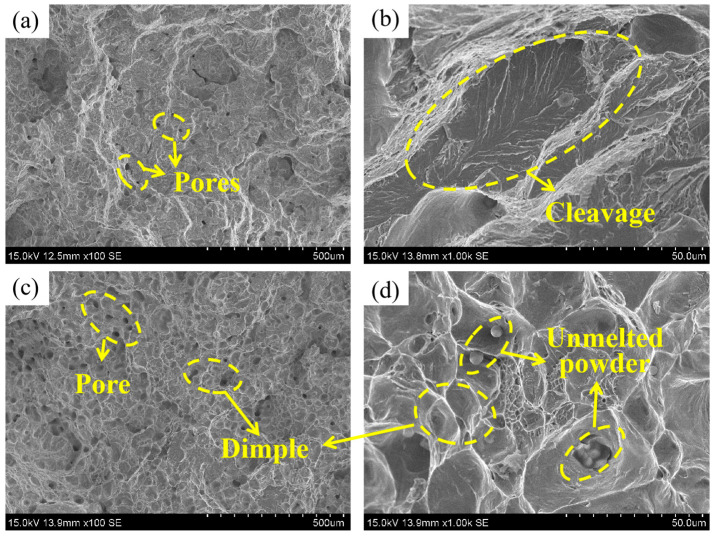
SEM images of the tensile fracture of sintered parts at different temperatures: (**a**,**b**) 1300 °C, (**c**,**d**) 1390 °C.

The tensile strength results presented in Figure 11c reveal a noteworthy increase in tensile strength, rising from 567.53 MPa to 770 MPa, representing a 35.68% increase as temperature escalates from 1300 °C to 1390 °C. Figure 11d shows the stress–strain curves of the sintered parts at different temperatures. These curves indicate that the 15-5PH steel parts experienced fracturing with a relatively small plastic deformation. Tensile strength and relative density are closely related. The part sintered at 1300 °C contains more pores, which act as a source of cracks during the tensile process, making the material unable to withstand too high of a load before fracture. As the temperature rises, the internal pores of the part gradually shrink or even close and the porosity decreases. The degree of pore spheroidisation is high and mostly small, so these pores require higher stress conditions to become crack sources, leading to the specimen being able to withstand higher loads. The fracture morphology of sintered parts at different temperatures is shown in Figure 13. Figure 13a shows a sample sintered at 1300 °C with many river-like patterns on the fracture surface, which is a typical appearance of a brittle fracture. The fracture morphology of the sintered part at 1390 °C (Figure 13b) shows that it still contains internal pores. It is noteworthy that in addition to the brittle fracture characteristics, there are also a large number of dimple-like micropores on the fracture surface at 1390 °C. Local magnification of the sintered parts at 1390 °C shows the presence of unmelted powders in the dimple-like micropores. These powders detach from the matrix under external forces to form micropores and gradually grow to form dimples.

## 4. Conclusions

This work has successfully developed 15-5PH stainless steel granules that can be used for metal FDM. Printing, debinding and sintering processes of metal FDM were investigated using these granules as a raw material. Metal parts were fabricated using low-cost 3D printing technology. The main conclusions of the research are as follows:

1. The 15-5PH stainless steel powder is evenly distributed in the granules without agglomeration. The MFR of the granule materials is sensitive to temperature changes, and the fluidity of the granules is the best at 285 °C. The selection of the nozzle diameter and the adaptability of the printer to the viscosity of granules are key to successful printing. The condition of the fan at the nozzle determines the surface quality of the part. The optimum printing parameters are a nozzle diameter 0.8 mm, an extrusion multiplier 180% and the fan shut off at the nozzle.
2. Solvent debinding removes soluble components from green parts and provides a pathway for gas diffusion during the thermal debinding process. The solvent debinding rate increases continuously with increasing debinding temperature and time. The debinding rate reaches its maximum at a temperature of 75 °C for 24 h, which is 98.7%. At the same temperature, the debinding rate increment gradually decreases and eventually stabilizes over time. During the thermal debinding process, sintering necks form between the metal powders, preserving the part’s structural integrity. All of the binder was removed and the weight loss was about 10% after debinding.
3. The relative density of sintered parts experiences a steady rise with increasing sintering temperature, progressing from 87.26% at 1300 °C to 95.83% at 1390 °C. The microstructure indicates that the parts sintered at 1300 °C contain many pores with large sizes. And the number and size of pores decrease significantly at 1390 °C. The dimensional shrinkage of the sintered parts remains uniform in the X-Y-Z directions. The shrinkage amplifies as the sintering temperature rises, with the range of shrinkage varying from 13.26% to 19.58% within the temperature range of 1300 °C to 1390 °C.
4. The hardness and tensile strength of sintered parts increase with increasing temperature, which is mainly related to the density of the part. The hardness of the sintered parts is 87.48 ± 1.03 HRBW at 1300 °C, and it does not change significantly between 1375 °C and 1390 °C. The tensile strength of the sintered parts increases from 567.53 MPa at 1300 °C to 770 MPa at 1390 °C, an increase of 35.68%. The fracture surface of the sintered parts at 1300 °C shows many dissociated sections, while the fracture surface at 1390 °C shows many dimples. The 15-5PH steel parts show brittle fracture with almost no plastic deformation.

This work achieves the low-cost additive manufacturing of metal parts, which has great advantages in the production of small batches and personalised parts. However, the mechanical properties of parts produced by metal FDM need to be improved, and better mechanical properties are expected to be achieved by adjusting the FDM printing parameters and optimising the debinding and sintering processes. Future research directions could focus on investigating printing parameters and changing sintering methods, such as using vacuum hot pressing sintering, microwave sintering and other sintering methods to improve the mechanical properties of materials. The sintered part can also be subjected to heat treatments such as solution and aging to improve the mechanical properties and expand its application scenarios.

## Figures and Tables

**Table 1:** Chemical compositions of 15-5PH SS powders versus national standards (wt%).

Elements	C	Si	Mn	S	P	Cr	Ni	Cu	Nb	Fe
ASTM	≤0.07	≤1.00	≤1.00	≤0.015	≤0.03	14.0–15.5	3.5–5.5	2.5–4.5	0.15–0.45	Bal
15-5PH	0.015	0.59	0.52	0.004	0.03	15.22	3.98	3.85	0.35	Bal

**Table 2:** Composition and proportion of 15-5PH granular feedstock.

Feedstock	15-5PH Powder	TPE	MAH-g-PP
Content (wt.%)	90	7	3

**Table 3:** Mass change after different debinding steps relative to the mass of green part.

Green Part mass/g	Solvent Debinding/(Δm,%)	Thermal Debinding/(Δm,%)	Total/(Δm,%)
10.7822	6.91%	3.16%	10.07%
10.7069	6.91%	3.19%	10.10%
10.6765	6.86%	3.17%	10.03%

## Data Availability

The data in this work are available from the corresponding authors on resonable request.
